# Psychosocial distress in outpatients with cancer: influence of demographic and medical factors on psychosocial distress and the perceived need of psycho-oncological support

**DOI:** 10.3389/fpsyg.2025.1747472

**Published:** 2026-01-14

**Authors:** Hannah Zingler, Lara Dreismann, Pia Hummels, Tanja Zimmermann

**Affiliations:** 1 Clinic for Psychosomatics and Psychotherapy, Hannover Medical School, Hannover, Germany; 2 Department of Stem Cell Transplantation, University Medical Center Hamburg-Eppendorf, Hamburg, Germany

**Keywords:** cancer outpatients, perceived need, psycho-oncological support, psycho-oncology, psychosocial distress

## Abstract

**Introduction:**

Psychosocial distress in cancer patients can have a significant impact on quality of life and adherence to treatment. Psychosocial distress is often systematically recorded in an inpatient setting. Psychosocial distress is also assessed in outpatient oncological care using psycho-oncological screening. However, there is currently little evidence that considers the psychosocial distress, the perceived need for support and the disease-related sociodemographic factors in outpatient cancer patients.

**Methods:**

In this cross-sectional study, routine data from *N* = 868 outpatient cancer patients were analyzed. Psychosocial distress was assessed using the Distress Thermometer (DT) and specific problem areas were identified using the Problem Checklist (PCL). Sociodemographic and medical factors were examined with regard to their influence on the experience of distress and the perceived need for psycho-oncological support.

**Results:**

46.1% of cancer patients reported high distress (DT ≥ 5) and 10% indicated a perceived need for psycho-oncological support. Younger age, female gender, and the first year after diagnosis were significantly associated with high distress. The most common physical and psychosocial problems included skin problems, exhaustion, sleep disorders, pain, worries, and anxiety.

**Discussion:**

The results underscore the need for standardized distress screening in outpatient care, in order to identify need for support at an early stage and provide targeted interventions. Future studies should examine the factors that may influence utilization in more detail so that barriers can be further reduced and psycho-oncological interventions can be offered in an outpatient setting in a manner that is tailored to needs and requirements.

## Introduction

An increasing number of cancer cases are being treated exclusively on a semi-inpatient or outpatient basis (Baumann and Schmitz, 2016), and shorter hospital stays are also leading to a faster transition to the outpatient sector (Bundesamt., D.S, 2023). As a result, cancer patients often seek outpatient clinics, care centers, and specialist practices for their diagnosis, treatment, and follow-up care (Hermes-Moll and Heidt, 2019). For many patients, this offers a better quality of life and greater social/family well-being compared to inpatient treatment (Hinz et al., 2018; Martino et al., 2018). The outpatient care structures for patients and their relatives however still have considerable room for improvement compared to the inpatient sector (Singer et al., 2017). In the outpatient setting in particular, referrals to psycho-oncology and utilization by patients appear to be delayed (Rausch et al., 2023). In addition, psychosocial distress and the perceived need for psycho-oncological support are less frequently systematically recorded in outpatient practices than in the inpatient sector, where psycho-oncological screening is a certification-relevant indicator for certified oncology and organ cancer centers (Onkozert, 2022).

In this context, needs-based and target-oriented psycho-oncological care is essential, for example to prevent mental health problems from becoming chronic. Significant psychosocial distress increases the risk of developing a clinically significant mental disorder, with corresponding limitations and consequences (Mehnert-Theuerkauf et al., 2023). Psycho-oncological interventions have long been proven effective in reducing psychosocial stress and improving quality of life (Andersen et al., 2008). Validated screening procedures are effective in identifying these stresses and are therefore anchored in both certification criteria and guidelines (National Comprehensive Cancer Network, 2020). The psychosocial distress is determined on the basis of subjectively reported distress when a threshold value defined by screening procedures is exceeded (Stengel et al., 2021). The perceived need for support is also assessed based on the patient’s subjective indication of a desire for psycho-oncological support (Dinkel et al., 2024).

A large number of studies in inpatient settings have shown that cancer is associated with high psychosocial distress (Meggiolaro et al., 2016; Mehnert et al., 2018; Peters et al., 2020). Approximately 50% (Mehnert et al., 2018) to 65% (Peters et al., 2020) of inpatients with cancer report significantly high psychosocial distress. A narrative summary of the stress areas by Shivappa et al. (2024) shows the wide range of stress experienced by cancer patients. There are comparatively fewer studies on the burden on outpatient cancer patients, partly due to the frequent lack of routine data. In outpatient care, a 2018 study found that 49.7% of patients receiving outpatient treatment showed significant distress, which is no different from the prevalence in the inpatient sector (Mehnert et al., 2018). Outpatients with cancer have the greatest need for support in the area of psycho-oncology, followed by needs related to the healthcare system and information needs. On average, they report 12 problems in the areas of practical, emotional, family, spiritual, and physical problems (Rosenberger et al., 2012).

In order to identify patients with higher risk of psychosocial distress, possible risk factors and influencing factors need to be identified. In general, female gender, younger age, and advanced stages of cancer may be associated with higher psychosocial distress (Peters et al., 2020). There are heterogeneous findings regarding the treatment goal, somatic factors, and their influence on stress (Mitchell et al., 2011a; Riedl and Schüßler, 2022), with stress being particularly high in the first months after diagnosis (Lee and Hong, 2025) and patients with gynecological tumors have the highest stress levels and prostate cancer patients the lowest (Mehnert et al., 2018). In addition, there are factors that have a positive effect on the use of psycho-oncological support. In outpatients, younger age, higher educational attainment, higher psychosocial stress, low emotional well-being, lack of social support, and recommendation by the treating oncologist were found to be predictors of the desire for and the use of support (Riedl et al., 2018; Frey Nascimento et al., 2019). In patients transitioning from inpatient to outpatient uro-oncological treatment, younger age and higher anxiety levels were also found to be predictors of subsequent use of outpatient support (Fugmann et al., 2025).

However, there is a lack of studies that examine psychosocial stressors and perceived needs as well as risks and influencing factors in large populations with mixed entities. In Germany, too, there are no studies that focus on the outpatient oncology care system. Consequently, the present study aims to (a) analyze the demand based on psychosocial distress and the perceived need among cancer patients of various entities receiving outpatient treatment in Germany, and (b) identify sociodemographic and medical factors influencing psychosocial distress, the perceived need for psycho-oncological support, and individual problem areas.

## Methods

### Study design

The retrospective observational study was conducted using cross-sectional routine data from outpatient distress screening at the Medical University of Hanover and was collected between July 2016 and July 2024. As part of the certification process, it is specified that patients in outpatient care receive the questionnaire once every 3 months. Criteria for receiving the screening questionnaire were a confirmed cancer diagnosis and sufficient German language skills and cognitive abilities to complete the questionnaire independently. Completion of the screening was voluntary and could be refused without giving reasons, although no data on the number and reasons for refusal are available as part of routine clinical practice. The completed screening questionnaires were combined with information on age, gender, outpatient clinic, date of initial diagnosis, cancer entity according to the International Classification of Diseases and Related Health Problems (ICD-10) from the hospital information system and then anonymized for further analysis. Cases with multiple missing information were excluded from the analysis.

### Measuring instruments

#### Distress thermometer and problem list

To assess the need for psycho-oncological support, the Distress Thermometer (DT) was used in combination with the Problem Checklist (PCL) of the National Comprehensive Cancer Network (NCCN) (Mehnert et al., 2006; Weis et al., 2022). The DT is a valid, easy-to-use ultra-short screening tool that measures the acute stress experienced by cancer patients on a scale of 0 to 10. The corresponding item is: *“Please circle the number (0–10) that best describes how distressed you have felt in the last week, including today (0 = not at all distressed; 10 = extremely distressed).”* The cut-off value for high distress is ≥ 5, values ≥ 8 are interpreted as *“severe distress”* (Mitchell et al., 2011b).

The problem list supplements the DT with more detailed information by summarizing specific problem areas into five superordinate problem areas with a total of 36 items [practical (5), family (2), emotional (6), spiritual/religious (2), physical (21)] using a binary yes/no question.

The perceived need is also assessed in a separate question using a binary yes/no query: *“Would you currently like counseling or support from psycho-oncology?*”

The screening questionnaires were given to patients in paper form during their appointments at the respective outpatient clinics and were completed on site.

### Statistical analysis

The statistical analysis of the data was performed using IBM SPSS software, version 29.0.0. The Shapiro–Wilk test was used to verify the distribution assumptions. The data were not normally distributed but the sample size was sufficient. The categorization of age groups is based on the categories proposed in a study by Meeker et al. (2017). Tumor entities were categorized according to the official ICD-10 classification (DIMDI (Deutsches Institut für Medizinische Dokumentation und Information), 2020), and disease stage was classified according to the official group classification of the Union for International Cancer Control (UICC). Unpaired t-tests and one-factor ANOVAs with subsequent Tukey post-hoc tests were calculated for group comparison. In cases where Levene’s tests showed that the assumption of variance homogeneity was violated, the more robust Welch ANOVA with subsequent Games-Howell *post hoc* tests was interpreted. For the analysis of risk factors, odds ratios were calculated using cross tables. To ensure voluntary participation, not all participants completed all parts of the questionnaire, which is why the individual sample sizes may vary. Significance was set at *α* = 0.05.

## Results

### Sample

A total of *N* = 868 screenings of outpatients with cancer were available, of whom 58.8% (*n* = 510) were male (see Table 1). The age range was between 27 and 97 years (*M* = 64.46, *SD* = 14.07).

**Table 1:** Sociodemographic and medical data of the sample (*N* = 868).

Sample characteristics	Total sample (*N* = 868)	Female (*n* = 353)	Male (*n* = 510)	*p*
Average age in years	64.46	64.81	64.21	0.544
(SD, range)	(14.07, 27–97)	(14.84, 31–90)	(13.52, 27–97)	
Marital status (*n*, %)				<0.001
Married	486 (61.8)	177 (54.3)	307 (67.2)	
Single/living alone	140	53 (16.3)	86 (18.8)	
Widowed	83 (10.5)	67 (20.6)	16 (3.5)	
In a committed relationship	78 (9.0)	29 (8.9)	48 (10.5)	
Current employment status (*n*, %)				<0.001
Retired	467 (62.1)	209 (67.6)	256 (58.3)	
Employed	237 (31.5)	72 (23.3)	164 (37.4)	
Looking for work	23 (3.1)	8 (2.6)	15 (3.4)	
Housework	17 (2.3)	16 (5.2)	0	
Other	8 (1.1)	4 (1.1)	4 (0.9)	
Average number of children				0.981
(SD, range)	1.44 (1.15, 0–7)	1.44 (1.15, 0–7)	1.44 (1.51, 0–6)	
Of which minors	1.49 (*n* = 88)	37 (1.22)	51 (1.69)	0.135
(SD, range)	(0.91, 1–6)	(0.58, 1–3)	(1.05, 1–6)	
Cancer diagnoses (*n*, %)				<0.001
Skin (C43-C44)	554 (63.8)	253 (72.8)	336 (66.5)	
Malignant neoplasms without localization (C76-C80)	87	29 (8.3)	61 (12.1)	
Digestive organs (C15-C26)	60 (6.9)	7 (2.0)	6 (1.2)	
Respiratory organs (C30-C39)	43	17	28	
Genital organs (C51-C63)	29	9 (2.4)	23 (4.6)	
Urinary organs (C64-C68)	23 (2.6)	12 (3.5)	13 (2.6)	
Lymphatic, hematopoietic, or related tissue (C81-C96)	22 (2.5)	5 (1.4)	18	
Mesothelial tissue, soft tissue (C45-C49)	7 (0.8)	1 (0.3)	5 (1.0)	
Eye, brain, central nervous system (C69-C72)	6 (0.7)	4 (1.1)	3 (0.6)	
Chest (C50)	1 (0.3)	1 (0.3)		
Abnormal findings without diagnosis (R90-R94)	1 (0.3)	1 (0.3)		
Average time since diagnosis in months				0.800
(SD, range)	43.02 (52.72, 0–394)	42.47 (44.94, 0–379)	43.40 (57.53, 0–394)	
Average number of diagnoses				0.109
(SD, range)	2.06 (1.07, 1–7)	1.99 (1.06, 1–7)	2.10 (1.07, 1–6)	
UICC classification (*n*, %)				0.902
Stage I	54 (7.4)	23	31 (7.2)	
Stage II	45 (6.2)	13 (4.3)	32 (7.5)	
Stage III	231 (31.6)	106 (35.1)	125 (29.1)	
Stage IV	401 (54.9)	160 (53.0)	241 (56.2)	
Average psychosocial distress (DT)				0.040
(SD, range)	4.15 (2.64, 0–10)	4.38 (2.59, 0–10)	3.98 (2.66, 0–10)	
Perceived need of PSO				0.272
*n* (%)	87 (10.0)	40 (12.3)	44 (9.8)	

SD, Standard deviation; UICC, Union for International Cancer Control; PSO, Psycho-oncology; DT, Distress thermometer.

### Psychosocial distress and perceived need as well as problem areas of outpatients with cancer

46.1% (*n* = 347) of patients reported clinically significant psychosocial distress (DT cut-off value ≥5), including 13.1% (*n* = 99) who reported very high psychosocial distress (cut-off ≥8).

In the entire sample, 10% of patients (*n* = 87) stated that they would like to receive support from psycho-oncology (perceived need).

The most frequently reported physical problems in the problem list were dry/itchy skin (39.7%), exhaustion (37.7%), sleep problems (32.0), and pain (31.8%), while the most frequently reported psychological problems were worries (33.6%) and anxiety (31.5%) (see Table 2). On average, patients reported 4.65 (*SD* = 4.35, [0,24]) of the 34 problems listed. 15.5% (*n* = 126) of patients reported experiencing none of the symptoms. Half of the participants reported 3 or more problems (*Mdn* = 3.00).

**Table 2:** Frequency of problems (PCL) in the total sample (*N* = 868) and for female (*n* = 353) and male (*n* = 510) patients.

Problems	Total sample		Female		Male	
	*n*	%	*n*	%	*n*	%
Practical problems						
Transportation	38	4.9	21	6.5	17	3.7
Work/school	35	4.5	15	4.7	19	4.2
Housing	29	3.7	16	4.9	13	2.8
Insurance	13	1.7	7	2.2	6	1.3
Childcare	5	0.7	3	1.0	2	0
Family problems						
Family problems: Partner	29	3.8	9	2	20	4.4
Family problems: Children	17	2	9	2	8	1.8
Emotional problems						
Fears	249	31.5	132	40.0	116	25.3
Concerns	263	33.6	102	31.5	160	35.0
Nervousness	183	23.	93	28.4	87	19.1
Sadness	157	20	99	30.5	57	12.5
Loss of interest in daily activities	95	12	4	12	5	11.7
Depression	78	10.0	38	11.6	39	8.6
Spiritual problems						
In relation to God	19	2.6	6	2.0	13	3
Loss of faith	15	2	4	1.3	11	2.5
Physical problems						
Dry/itchy skin	316	39.7	15	46	162	34.9
Exhaustion	299	37.7	140	42.9	157	33.8
Sleep	253	32.0	122	37.5	129	27.8
Pain	251	31.8	139	41.9	111	24.4
Exercise/mobility	238	30.1	109	33.5	127	27.5
Tingling in hands and feet	148	18.7	77	23.3	71	15.5
Breathing	127	15.9	56	17.0	71	15.3
Digestive disorders	90	11.4	39	11.8	49	10.7
Food/nutrition	89	11.3	46	14.1	43	9.3
Nausea	75	9.5	37	11.5	37	8
Constipation	75	9.5	35	10.7	4	8.7
Swollen/bloated	69	8.8	36	11.1	32	7.0
Diarrhea	69	8.7	34	10.4	34	7.3
Washing/dressing	65	8.2	30	9.2	34	7.3
Changes in urination	65	8.1	30	9.1	35	7.5
Sexual problems	59	7.	14	4.5	44	9.8
Memory/concentration	52	18.8	26	22.4	25	15.7
Inflammation in the mouth area	41	5.	2	6.4	20	4.3
Appearance	33	4.2	16	5.0	17	3.7
Dry/stuffy nose	13	16.7	68	21.1	62	13.4
Fever	12	1.5	9	2.8	3	0.6

Percentages always in relation to valid cases depending on category, sample size varies between *N* = 116 (item memory/concentration was only added during the course of the study) and *N* = 799.

### Factors influencing psychosocial distress, perceived need, and problem areas

#### Psychosocial distress

No significant differences in psychosocial distress were found with regard to work situation [*F*(4, 692) = 1.95, *p* = 0.101], marital status [*F*(3, 725) = 1.59, *p* = 0.191], patients with or without children (95% CI [−0.17; 0.69], *t*(322.96) = 1.21, *p* = 0.227), number of children [Welch test *F*(1, 291,734) = 1.18, *p* = 0.278], of which minors [*F*(4, 11.57) = 1.67, *p* = 0.155], UICC stage [*F*(3, 634) = 1.42, *p* = 0.235], and number of cancer diagnoses [*F*(6, 733) = 1.21, *p* = 0.301].

**Figure 1:**
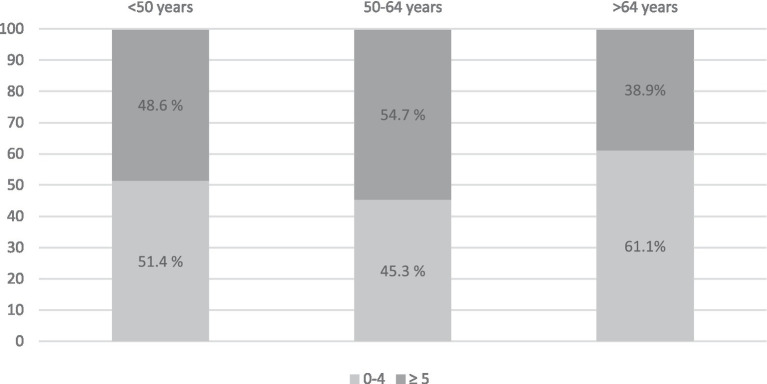
Frequency (in %) of distress levels measured with the distress thermometer in the age groups.

Significant differences were found for gender, age group, cancer diagnoses, time since diagnosis, and desire for support. Women reported significantly higher distress (*n* = 156, 49.2%) than men (*n* = 190, 43.8%) (*M*_female_ = 4.38, *SD* = 2.59; *M*_male_ = 3.98, *SD* = 2.66, 95% CI [0.02, 0.78], *t*(749) = 2.06, *p* = 0.040). The age groups (see Figure 1) differed statistically significantly in terms of average distress scores [*F*(2, 80.156) = 11.81, *p* < 0.001] in the middle age group (50–64 years, *M* = 4.73, *SD* = 2.65) and older age (≥65 years, *M* = 3.72, *SD* = 2.09, *p* < 0.001, 1.007, 95% CI [0.52, 1.50]).

There was a statistically significant difference in cancer diagnoses [Welch test *F*(9, 64.39) = 10.44, *p* < 0.001] in terms of distress, with patients diagnosed with malignant neoplasms of the respiratory organs and other intrathoracic organs (C30-C39) showing particularly high distress scores. Tukey HSD post-hoc tests showed statistically significant differences (*p* < 0.001) in the distress scores of these patients (*M* = 7.33, *SD* = 2.11) compared to patients diagnosed with malignant neoplasms of the lip, oral cavity, and pharynx (C00–C14) (*M* = 3.76, *SD* = 0.72, [1.50, 6.04] *p* < 0.001), malignant neoplasms of the digestive organs (C15–C26) (*M* = 3.49, *SD* = 0.52[1.82, 5.17] *p* < 0.001), malignant neoplasms of the skin (C43–C44) (*M* = 3.42, *SD* = 0.42, −3.421, [2.10, 4.74] *p* < 0.001), malignant neoplasms of female genital organs (C51–C58) (C15–C26) (*M* = 4.04, *SD* = 1.03[0.76, 7.32] *p* = 0.004), malignant neoplasms of the male genital organs (C60–C63) (C15–C26) (*M* = 2.48, *SD* = 0.70 [0.25, 4.71] *p* = 0.016), malignant neoplasms of urinary organs (C64–C68) (C15–C26) (*M* = 2.39, *SD* = 0.75 [0.02, 4.75] *p* = 0.046), malignant neoplasms of unspecified, secondary or unspecified sites (C76–C80) (*M* = 3.26, *SD* = 0.49, [1.69, 4.82] *p* < 0.001) and malignant neoplasms of lymphatic, hematopoietic, and related tissues (C81–C96) (*M* = 3.76, *SD* = 0.69 [1.58, 5.97] *p* < 0.001).

Patients in the first year after diagnosis showed statistically significantly higher distress scores (*M* = 4.50, *SD* = 2.56) than patients whose diagnosis was more than 1 year ago (*M* = 3.96, *SD* = 2.67, 95% CI [0.14, 0.93], *t*(751) = 2.65, *p = 0*.008).

#### Problem areas

Women most frequently reported dry/itchy skin (46.5%), exhaustion (42.9%), and pain (41.9%), while men reported dry/itchy skin (34.9%), worry (35.0%), and exhaustion (33.8%) (see Table 3). There is a statistically significant difference in the number of emotional problems reported between the sexes, with female patients reporting an average of 0.34 more problems (*M =* 1.37, *SD* = 1.69) than male patients (*M =* 1.03, *SD* = 1.49, 95% CI [0.11, 0.57], *t*(586.62) = 2.82, *p* = 0.005, *d* = 0.21). There was also a statistically significant difference in the number of physical problems reported, with female patients reporting an average of 1.22 more problems (female: *M* = 3.65, *SD* = 3.15; male: *M* = 2.43, *SD* = 2.62, 95% CI [0.44, 2.01], *t*(173.61) = 3.09, *p* = 0.002, *d* = 0.42).

**Table 3:** Gender differences in the Problem Checklist (PCL).

Problem areas	Female		Male		*t*	*df*	*p^a^*	*d*
	*M*	*SD*	*M*	*SD*				
Practical problems	0.16	0.48	0.11	0.37	1.38	541.69	0.169	0.11
Family problems	0.05	0	0	0.25	−0.22	745	0.824	-
Emotional problems	1.3	1.69	1	1.49	2.82	586.62	0.005	0.21
Spiritual problems	0.03	0.21	0.06	0.30	−1.35	730.99	0.178	−0.10
Physical problems	3.65	3.15	2.43	2.62	3.09	173.61	0.002	0.42

^a^Significance level *p* < 0.05.

With regard to age groups, there is a shift in the three most frequently mentioned problems with increasing age, from a dominance of psychological and physical problems such as worry (56.6%) exhaustion (50.5%) and anxiety (44.4%) among those under 50 to a predominance of physical problems such as dry/itchy skin (40.2%), pain (31.2%) and problems with movement and mobility (31.1%) among those aged 65 and over. In the overarching problem areas (see Table 4), statistically significant differences were found in the number of practical problems reported [Welch test *F*(2, 256.60) = 3.69, *p* = 0.026], family problems [Welch test *F*(2, 245.62) = 4.62, *p* = 0.011], emotional problems [Welch test *F*(2, 254.12) = 13.37, *p* < 0.001], and spiritual problems [Welch test *F*(2, 214.01) = 4.75, *p* = 0.010].

**Table 4:** Differences between age groups in terms of the number of different problems according to the area of the Problem Checklist (PCL).

Problem areas	Age groups						*F*	*df1*	*df2*	*p^a^*
	<50		50		≥65					
	*M*	*SD*	*M*	*SD*	*M*	*SD*				
Practical problems	0.25	0.60	0.13	0.41	0.09	0.36	3.69	2	256.60	0.026
Family problems	0.11	0.32	0.06	0.30	0.03	0.18	4.62	2	245.62	0.011
Emotional problems	1.61	1.62	1	1	0.89	1.38	13.37	2	254.12	0.001
Spiritual problems	0.18	0.57	0	0.12	0.03	0.21	4.75	2	214.01	0.010
Physical problems	3.27	2	3.01	3.0	2.72	2.79	0.56	2	100.73	0.571

^a^Significance level *p* < 0.05, two-tailed.

### Correlation between problem areas and psychosocial distress

Looking at the most frequently mentioned problems (dry/itchy skin, exhaustion, and worry), it can be seen that the presence of one (OR = 4.50, 95% CI [3.20, 6.34], *p* < 0.001) or two (OR = 2.92, 95% CI [2.04, 4.17], *p* < 0.001) of these symptoms, there is an increased likelihood of experiencing high psychological distress. When all three symptoms (dry/itchy skin, exhaustion, and worry) are present, the risk of high distress is almost nine times higher compared to patients who did not report these problems (OR = 8.76, 95% CI [4.41, 17.42], *p* < 0.001).

#### Perceived need

There was no statistically significant difference between the sexes, *t*(654.59) = 1.10, *p* = 0.272. There were also no statistically significant differences in the perceived need for psycho-oncological support with regard to the number of cancer diagnoses [Welch test *F*(4, 101.32) = 2.09, *p* = 0.088], patients with or without children [Welch test *F*(1, 263.18) = 3.13, *p* = 0.078], and the number of minor children [*F*(4, 693) = 1.67, *p* = 0.155].

A statistically significant difference was found for the age groups [Welch test *F*(2, 268.87) = 6.54, *p* = 0.002]. Middle-aged patients (50–64 years) (*M* = 1.15, *SD* = 0.36) significantly more likely to desire psycho-oncological support than older patients (*M* = 1.07, *SD* = 0.24, 0.08, 95% CI [0.02, 0.14], *p* = 0.004). Working patients (*M* = 1.16, *SD* = 0.02) also reported a statistically significant higher perceived need than retired patients (*M* = 1.08, *SD* = 0.27, 0.09, 95% CI [0.01, 0.16], *p* = 0.015).

The UICC stage [Welch test *F*(3, 103.30) = 3.92, *p* = 0.01] has a significant influence on perceived need for psycho-oncological support. Patients with UICC stage I (*M* = 1.72, *SD* = 0.45) significantly less often wanted psycho-oncological support than patients with stage III (*M* = 1.92, *SD* = 0.27, 0.72, 95% CI [−0.39, −0.01], *p* = 0.032). and stage IV (*M* = 1.91, *SD* = 0.29, 0.071, 95% CI [−0.38, 0.00], *p = 0*.05).

Patients who had been diagnosed within the first year (*M* = 1.83, *SD* = 0.37) significantly more often desired psycho-oncological support than those who had been diagnosed more than 12 months earlier (*M* = 1.92, *SD* = 0.28, *t*(424.18) = 3.29, 95% CI [−0.136, −0.034], *p* < 0.001). In addition, patients with high distress scores (≥5) (*M* = 1.79, *SD* = 0.41) reported more often a perceived need for psycho-oncological support than patients with low distress scores (0–4) (*M* = 1.97, *SD* = 0.17, 95% CI [0.13, 0.23], *t*(425.33) = 7.52, *p* < 0.001, d = 0.60).

### Correlation between psychosocial distress and the perceived need

**Figure 2:**
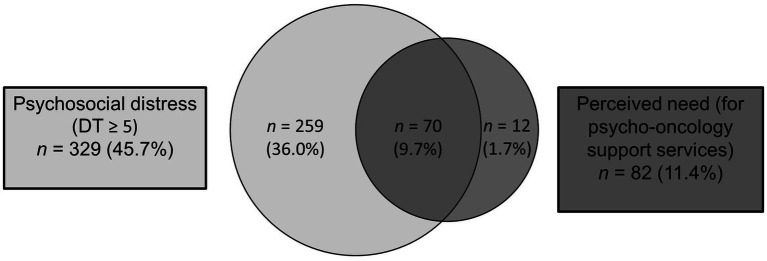
Comparison of psychosocial distress (DT value ≥5) and perceived need for psycho-oncological support.

*N* = 70 (8.1%) patients reported high distress levels and a perceived need for psycho-oncological support. *N* = 12 (1.4%) showed a perceived need without reporting psychosocial distress, and *n* = 259 (29.8%) patients reported high psychosocial distress but denied a perceived need (see Figure 2).

There was a statistically significant correlation between psychosocial distress and perceived need (*χ*^2^(1) = 58.69, *p* < 0.001, *φ* = 0.29). Patients who expressed a need for support (perceived need) showed statistically significantly higher levels of psychosocial distress (*M* = 6.57, *SD* = 2.25) than patients who did not express this need (*M* = 3.80, *SD* = 2.53, 95% CI [2.24, 3.30], *t*(109.24) = 10.36, *p* < 0.001).

## Discussion

The aim of the present study was to investigate psychosocial distress, the most common problem areas, and the perceived need for psycho-oncological support among outpatient cancer patients, as well as factors influencing psychosocial distress. Overall, 46.1% of patients reported high psychosocial distress (DT ≥ 5). 13.1% reported severe distress (DT ≥ 8). The results are thus consistent with previous findings in the outpatient setting, where 49.7% of patients treated on an outpatient basis showed significant distress (Mehnert et al., 2018). Lower prevalence rates were found by Riedl et al. (2018) in an outpatient radiation therapy cohort, where the stress rate was 20%, measured with the Hornheider screening instrument. Lehmann-Laue et al. (2019) showed slightly higher mean values in the DT of *M* = 5.8 in a study conducted in a cancer counseling center compared to the present study with *M* = 4.15 (Lehmann-Laue et al., 2019). In a comparable study by Peters et al. (2020) in which inpatients were surveyed using the same questionnaire, 65.9% reported increased distress. One possible explanation for the difference between inpatients and outpatients was provided by a study by Hinz et al. (2018), in which inpatients reported a significantly poorer quality of life compared to outpatients.

In the present outpatient sample, almost every second person feels psychologically distressed. This confirms that distress screening and psycho-oncological support are also essential for the holistic recovery of patients in an outpatient setting. However, only 10% of those affected also expressed a perceived need for psycho-oncological support. This is consistent with the findings of Wünsch et al. (2024), according to which only 11% of outpatient skin cancer patients actively expressed a desire for counseling. Other studies also show this discrepancy between the significant need and the subjective lack of need for support among cancer patients (Clover et al., 2015; Riedl et al., 2018; Wünsch et al., 2024). Clover et al. (2015) surveyed reasons for not taking advantage of psycho-oncological support services. The most common reasons given were the desire to cope with the stress themselves (46%), already receiving help (24%), and not considering their own stress to be serious enough (23%). Patients most frequently cite a lack of perceived need, a lack of information about psycho-oncology, logistical problems, a lack of trust in psychosocial services, fear of stigmatization, and a lack of recommendations from their primary care providers (Dilworth et al., 2014; Betker et al., 2025). These findings are reflected in the recommendation to combine screening procedures with information about support services and feedback on the screening results to patients, together with a personalized recommendation (Carlson et al., 2012; Frey Nascimento et al., 2019; Stengel et al., 2021; Dreismann et al., 2023). Dreismann et al. (2023) emphasize the importance of training the clinical team in screening procedures, communication regarding screening results, providing information about psycho-oncological support services and personalized communication to reduce barriers in support uptake.

In the present sample, younger patients (<50 years) and female patients showed significantly higher distress scores. These findings are consistent with previous studies (Carlson et al., 2004; Meggiolaro et al., 2016; Carlson et al., 2019; Peters et al., 2020; Erdoğan Yüce et al., 2021). However, differences were found depending on the cancer diagnosis. Patients with lung cancer reported the highest stress levels. Nevertheless, earlier studies also show heterogeneous findings regarding diagnosis-specific differences in distress experiences. There are assumptions about the general and individual influence of the intensity of the necessary treatment measures, the assessment of prognoses, and the degree of influence of the specific type of cancer on everyday life, as well as individual coping strategies or social support (Peters et al., 2020).

The present study identified the first year of cancer (up to and including 12 months after diagnosis) as a risk factor for high distress. This finding is consistent with previous studies and confirms the assumption that coming to terms with the diagnosis, lifestyle changes, and acute treatments are particularly stressful at the beginning and that early support services are very important (Erdoğan Yüce et al., 2021).

The report of higher stress levels among women was also associated with a significantly higher number of mental and physical problems in the PCL of female patients compared to male patients. However, gender differences should not be interpreted exclusively as a biological factor. Rather, psychosocial variables such as socialization, communication and expression of emotions, social support, gender-specific roles and expectations, health behavior, and socially influenced coping strategies could be influencing factors and explain a larger proportion of the gender discrepancy (Mehnert et al., 2018; Hoffer-Pober and Strametz-Juranek, 2020).

Overall, the average number of problems reported per patient (4.7 out of a possible 34) is moderate. In the study by Lehmann-Laue et al. (2019), an average of 14 problems per patient were reported, although the sample in this study consisted of patients at a psychosocial counseling center who had specifically sought psycho-oncological support. The most frequently reported problem in the entire sample were physical symptoms such as dry/itchy skin. Dry and itchy skin is a common side effect of chemotherapy and radiation therapy (Almeida et al., 2023). The data on other problem areas are similar to the findings of other research groups (Carlson et al., 2004; Grassi et al., 2011; Lehmann-Laue et al., 2019; Mehnert et al., 2018). People with multiple problems of this kind show up to nine times higher risk of high distress compared to patients without these problems.

An important research gap was a comparison between the psychosocial distress and the perceived need in outpatient oncology patients. The results show that patients with high distress significantly more often want psycho-oncological support (perceived need) than less distressed patients. Faller et al. (2016) also showed in earlier studies that 51.2% of patients with psychosocial distress also had a perceived need (Faller et al., 2016). The current findings therefore reaffirm the importance of conducting distress screenings for all patients, including those in outpatient care, and of offering psycho-oncological support as a basis for targeted psycho-oncological care.

### Strengths and weaknesses

One advantage of this study is the large sample of outpatients and the routine data collected in a practical setting. Nevertheless, the study has some limitations. Due to its cross-sectional design, no causal conclusions or statements can be made about individual changes and long-term developments in psychosocial distress. In addition, there is a lack of information about which patients had already received psycho-oncological support and whether their stress levels improved as a result. However, this would be of empirical interest due to the duration of outpatient therapy, the long-term need for psycho-oncological counseling, and the sometimes lifelong effects of cancer. A further limitation arises from the need to maintain voluntariness, anonymity, and consideration for the sensitive data of patients, which resulted in very large individual sample differences due to incompletely filled out questionnaires. Furthermore, the data was obtained from routinely collected questionnaires. This poses the difficulty that no conclusions can be drawn about the rejection rate or the reasons for non-participation. This is a common problem in routine data collection and could lead to data distortion. It cannot be ruled out that patients who refuse preventive screening do not also differ systematically from those who participate. Future studies could use a longitudinal design to investigate how distress and the need for support change over the course of the disease, as well as factors that prevent patients from seeking psycho-oncological support. In addition, the present sample was dominated by skin cancer patients, which limits the generalizability to all patient groups and must be taken into account when interpreting the results. Further studies should therefore also take into account psychosocial distress and the perceived need for support in different types of cancer.

## Conclusion

The present results offer important implications for psycho-oncological care in outpatient oncology. Given the high prevalence of psychosocial distress in the outpatient setting and the number of challenges they have to face (Arndt et al., 2021; Ernst et al., 2024), the findings underscore the need for standardized distress screening in outpatient cancer care as well, both in the long term and during all phases of cancer and treatment, as well as afterward. Routine assessment allows support needs to be identified at an early stage, addressed in a targeted manner, and accompanied appropriately. Risk factors such as younger patients, women, lung cancer patients, and patients in their first year of illness were identified. This calls for greater awareness of the affected risk groups among healthcare professionals. Against the backdrop of the current challenges facing the healthcare sector and outpatient care in particular, tailored intervention services such as specific group services for younger patients with high levels of distress could pool resources in the best possible way and actively reach affected patients. More flexible support formats in terms of time and location, such as online or telephone-based support services, could also increase take-up in outpatient care in particular if they are actively promoted and easily accessible (Wünsch et al., 2024).

## Data Availability

The raw data supporting the conclusions of this article will be made available by the authors, without undue reservation.
