# Correction: Psychosocial distress in outpatients with cancer: influence of demographic and medical factors on psychosocial distress and the perceived need of psycho-oncological support

**DOI:** 10.3389/fpsyg.2026.1852162

**Published:** 2026-05-05

**Authors:** Hannah Zingler, Lara Dreismann, Pia Hummels, Tanja Zimmermann

**Affiliations:** 1 Clinic for Psychosomatics and Psychotherapy, Hannover Medical School, Hannover, Germany; 2 Department of Stem Cell Transplantation, University Medical Center Hamburg-Eppendorf, Hamburg, Germany

**Keywords:** cancer outpatients, perceived need, psycho-oncological support, psycho-oncology, psychosocial distress

There was a mistake in Figure 2 as published. A recalculation revealed slight discrepancies in the values. The corrected Figure 2 appears below.

**Figure 2:**
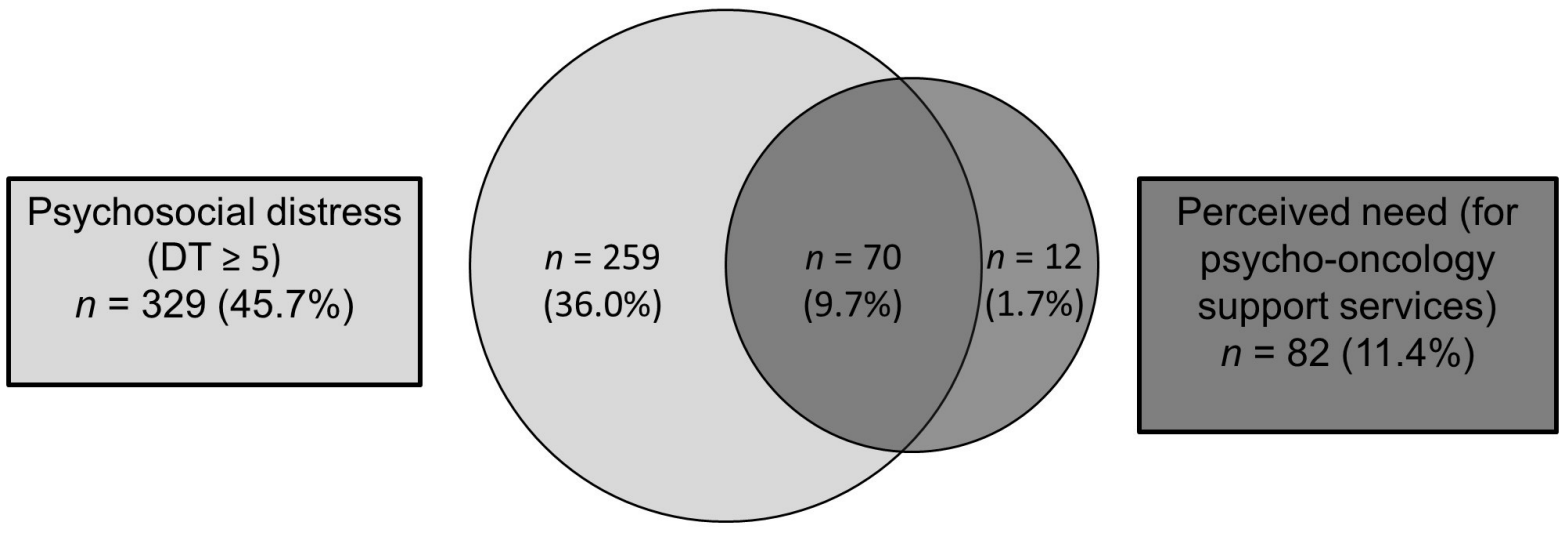
Comparison of psychosocial distress (DT value ≥5) and perceived need for psycho-oncological support.

The original version of this article has been updated.

